# Low-voltage, High-performance Organic Field-Effect Transistors Based on 2D Crystalline Molecular Semiconductors

**DOI:** 10.1038/s41598-017-08280-8

**Published:** 2017-08-10

**Authors:** Qijing Wang, Sai Jiang, Jun Qian, Lei Song, Lei Zhang, Yujia Zhang, Yuhan Zhang, Yu Wang, Xinran Wang, Yi Shi, Youdou Zheng, Yun Li

**Affiliations:** National Laboratory of Solid-State Microstructures, School of Electronic Science and Engineering, Collaborative Innovation Center of Advanced Microstructures, Nanjing University, Nanjing, 210093 China

## Abstract

Two dimensional (2D) molecular crystals have attracted considerable attention because of their promising potential in electrical device applications, such as high-performance field-effect transistors (FETs). However, such devices demand high voltages, thereby considerably increasing power consumption. This study demonstrates the fabrication of organic FETs based on 2D crystalline films as semiconducting channels. The application of high-κ oxide dielectrics allows the transistors run under a low operating voltage (−4 V). The devices exhibited a high electrical performance with a carrier mobility up to 9.8 cm^2^ V^−1^ s^−1^. Further results show that the AlO_x_ layer is beneficial to the charge transport at the conducting channels of FETs. Thus, the device strategy presented in this work is favorable for 2D molecular crystal-based transistors that can operate under low voltages.

## Introduction

Two-dimensional (2D) molecular crystals assembled through weak van der Waals forces are a promising class of materials for molecular packing and charge transport and exhibit significant potential for electronic applications^1–7^. Efforts have been devoted to the fabrications of organic field-effect transistors (OFETs) and p-n junctions that use ultrathin crystalline films grown on 2D atomic crystals, such as graphene, boron nitride, and MoS_2_. Recently, deposition of 2D molecular crystals on amorphous silicon oxide (SiO_2_) has been performed successfully through solution-based processes, which is compatible with current semiconductor manufacturing^8–17^. These OFETs also yielded high carrier mobility that are comparable to polycrystalline silicon. However, a high voltage (i.e., normally over 30 V) is necessary to operate such devices, which results in extra power consumption, because of the low dielectric constant of SiO_2_. Therefore, lowering the operating voltage is greatly important, particularly for portable and wearable electronics. An effective approach is to employ high-κ materials for the dielectric layers in transistor architectures, such that a low voltage can generate an adequate charge density in the conducting channel^18–21^. Therefore, producing low-operating-voltage and high-performance OFETs based on 2D molecular crystals using high-*κ* oxides as the dielectric layer, is noteworthy.

Herein, we demonstrate a low-voltage bottom-gate top-contact (BGTC) OFET that utilizes 2D molecular crystals as the conducting channel and AlO_x_ as the dielectric. The molecular crystals were solution-processed through a floating-coffee-ring-driven assembly according to the methods presented in our previous work^17^. Our devices can operate at a low gate bias of −4 V and exhibit a high carrier mobility (*μ*_FET_) up to 9.8 cm^2^ V^−1^ s^−1^, a large on/off ratio of 10^5^, and a small subthreshold swing of 160 mV dec^−1^. The results demonstrate that the proposed strategy has significant potential in fabricating low-voltage and high-performance OFETs employing high-*κ* dielectrics and 2D molecular crystals.

## Results

**Figure 1:**
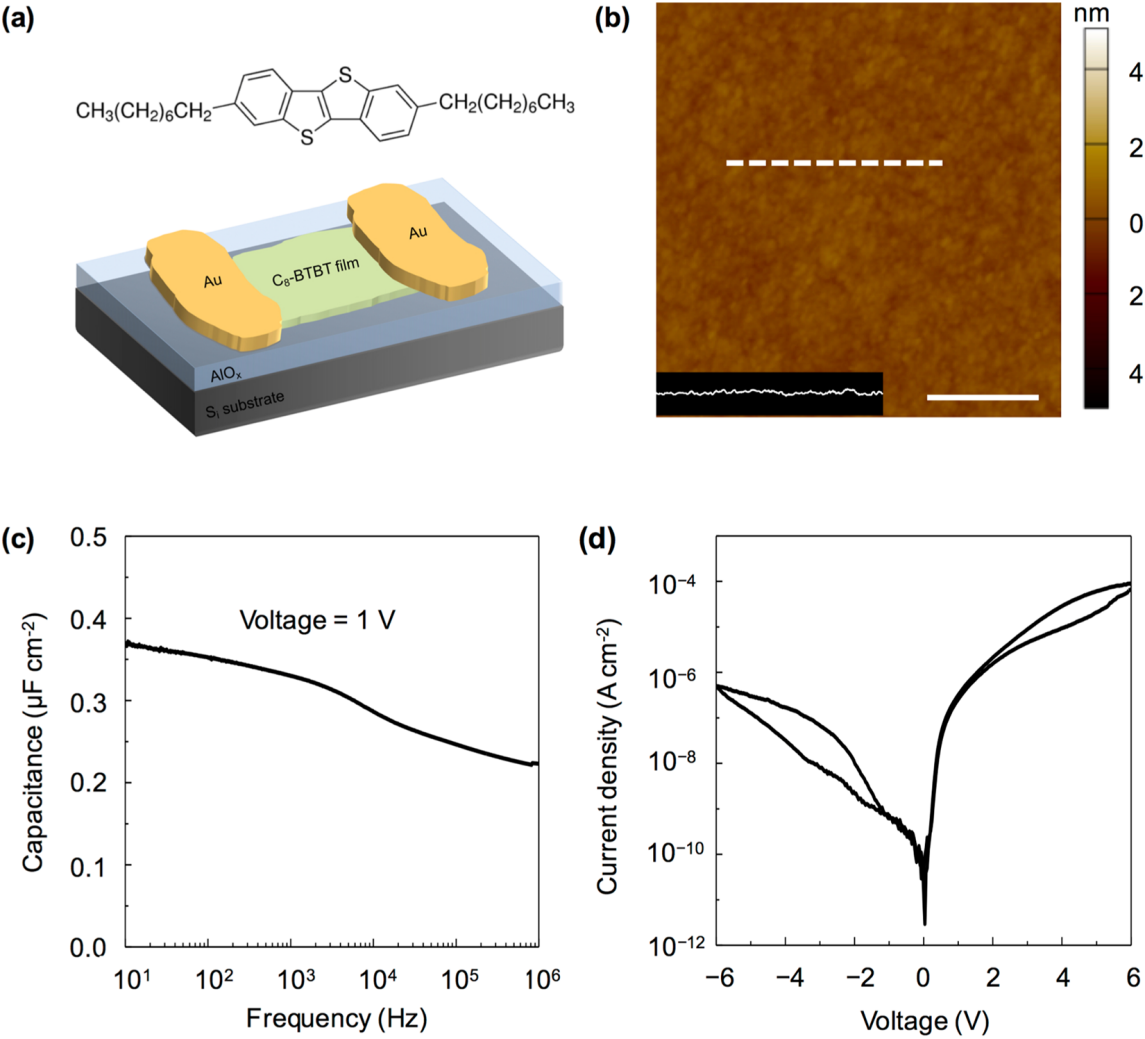
(**a**) Schematic of the BGTC transistor. (**b**) AFM morphology image of the AlO_x_ dielectric. Scale bar, 500 nm. (**c**) Capacitance per unit versus frequency (at a voltage of 1 V). (**d**) Gate leakage current versus voltage.

**Figure 2:**
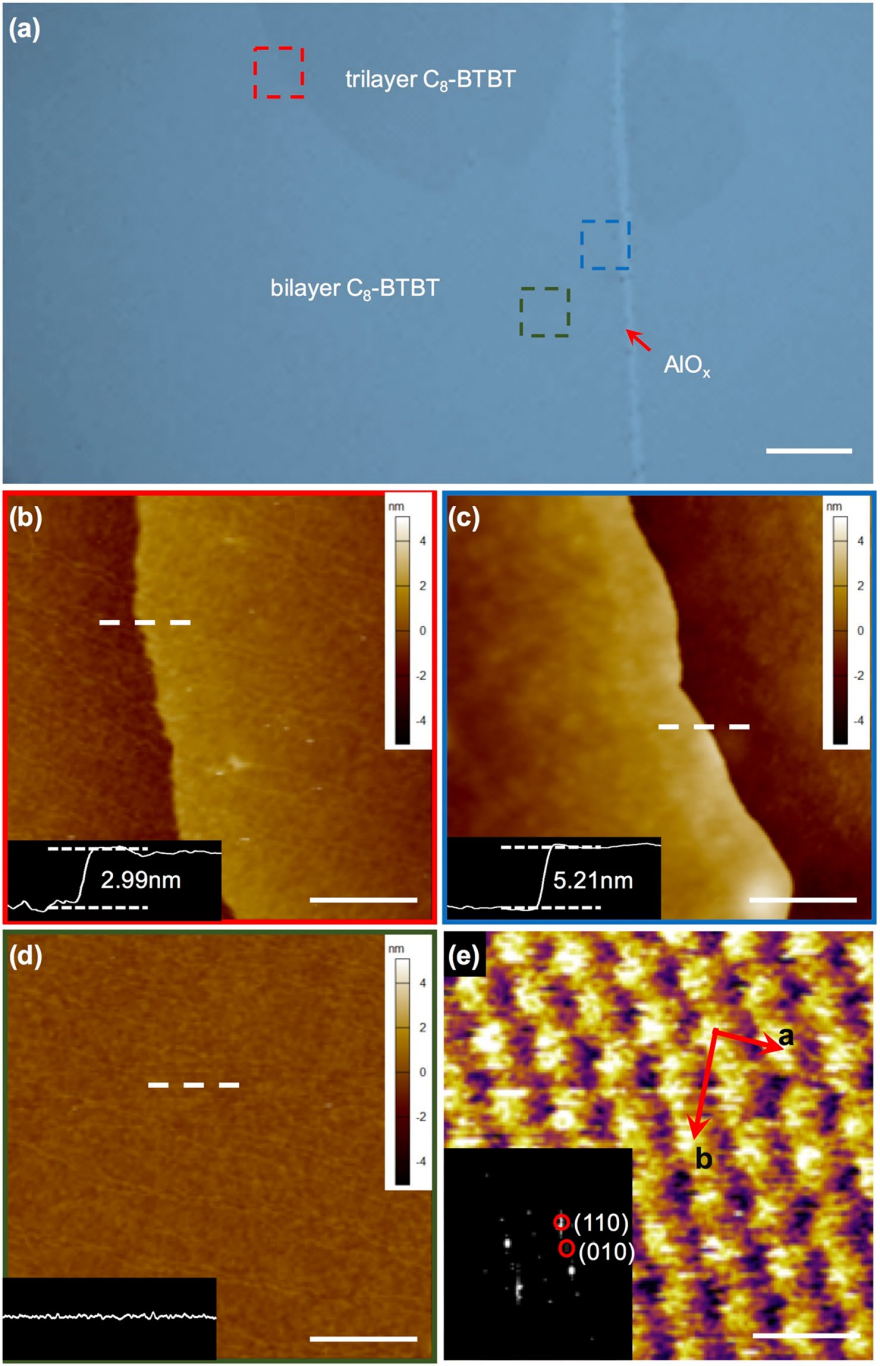
(**a**) Optical microscopy image of the 2D C_8_-BTBT crystalline films. The bilayer and trilayer are clearly observed. Scale bar, 20 μm. (**b**) AFM morphology image of the step between bilayer and trilayer films (red square in (**a**)). Scale bar, 500 nm. (**c**) AFM morphology image of the step between bilayer films and the substrate (blue square in (**a**)). Scale bar, 500 nm. (**d**) AFM morphology image of uniform bilayer films (brown square in (**a**)). Scale bar, 500 nm. (**e**) High-resolution AFM image of the bilayer C_8_-BTBT molecules on the AlO_x_/Si substrate. Scale bar, 1 nm. The inset shows the corresponding fast Fourier transforms of the AFM image with lattice indices.

The p-type small-molecule semiconductor of dioctylbenzothienobenzothiophene (C_8_-BTBT) exhibits a considerably high carrier mobility and thus has been used to fabricate the OFETs^22–24^. The highest occupied molecular orbital (HOMO) and the lowest unoccupied molecular orbital (LUMO) of C_8_-BTBT are −5.39 eV and −1.55 eV, respectively^25^. Figure 1a illustrates the BGTC structure of the transistor device that adopts thermally deposited AlO_x_ with a thickness of ~18 nm as the dielectric layer. Figure 1b shows the atomic force microscopy (AFM) image of the AlO_x_ dielectric, which illustrates a particularly smooth surface with a root-mean-square (RMS) roughness of 2.23 Å. The hydrophilicity of the AlO_x_ dielectric layer is enhanced through UV–ozone treatment. Improving the hydrophilicity is conducive to the subsequent solution-based process for molecular crystal growth. Besides, the surface roughness of UV-ozone-treated AlO_x_ is 2.75 Å (Supplementary Fig. S1). Given that the capacitance and gate leakage current are both critical to the gate dielectric in low-voltage OFETs, we employ an Au/AlO_x_/Si capacitor structure. The measured capacitance and dielectric constant are 0.37 μF/cm^2^ and ~9.0, respectively (measured at the voltage frequency of 10 Hz, Fig. 1c). Moreover, the AlO_x_ capacitance decreases from 0.37 μF/cm^2^ to 0.25 μF/cm^2^ when the frequency of the applied voltage increases from 10 Hz to 1 MHz. This effect is mainly due to the interfacial traps produced during the UV–ozone treatment. The dielectric capacitance of AlO_x_ exhibits negligible change when the applied voltage increases from −6 V to 6 V (Supplementary Fig. S2), and its leakage current is 10^−7^ A/cm^2^ when the applied voltage is −4 V (Fig. 1d). Therefore, the thermally deposited AlO_x_ can be used as a gate insulating layer because of its superior performance as a dielectric material. The coffee-ring-driven method (Supplementary Fig. S3) is utilized for the deposition of 2D films where C_8_-BTBT molecules assemble into 2D crystalline films with a large size of ~200 μm on the AlO_x_ surface (Fig. 2a)^17^. Supplementary Fig. S4 shows a deposited bilayer C_8_-BTBT film with a large size of several millimeters. The obtained molecular films consist of different C_8_-BTBT layers with step-and-terrace structures. Figure 2b and c show the AFM images of the two steps as marked by dotted squares in Fig. 2a, where the thicknesses are 2.99 and 5.21 nm, respectively. The molecules in the third layer are nearly perpendicular to the substrates with regard to the C_8_-BTBT molecular length. The previous results show that C_8_-BTBT molecules in first layer are more tilted to the substrate than that in upper layers, because the weak van der Waals interactions among the small molecules decrease rapidly from the dielectric surface to the upper molecular layers^6, 26^. The schematic illustration of C_8_-BTBT molecular packing is shown in Supplementary Fig. S5. Furthermore, the bilayer C_8_-BTBT exhibits uniform thin films with atomic smoothness (RMS roughness: 1.22 Å, Fig. 2d). The crystalline properties of the bilayer C_8_-BTBT are characterized through high-resolution AFM (Fig. 2e). More than 10 points are selected randomly for scanning by high-resolution AFM (Supplementary Fig. S6). The AFM images show nearly identical lattice constants, namely, *a* = 6.21 ± 0.16 Å, *b* = 8.12 ± 0.12 Å, and *θ* = 88.1 ± 1.4°. These results indicate that the bilayer C_8_-BTBT films each contain a crystalline phase with highly morphologic uniformity over a large area. Similar crystalline characteristics are also observed in the C_8_-BTBT trilayer (Supplementary Fig. S7). The crystalline properties of our molecular crystals are summarized in Supplementary Table S1. Therefore, the AlO_x_ layer formed through thermal evaporation facilitates the deposition of high-quality 2D C_8_-BTBT crystalline films.

## Discussion

**Figure 3:**
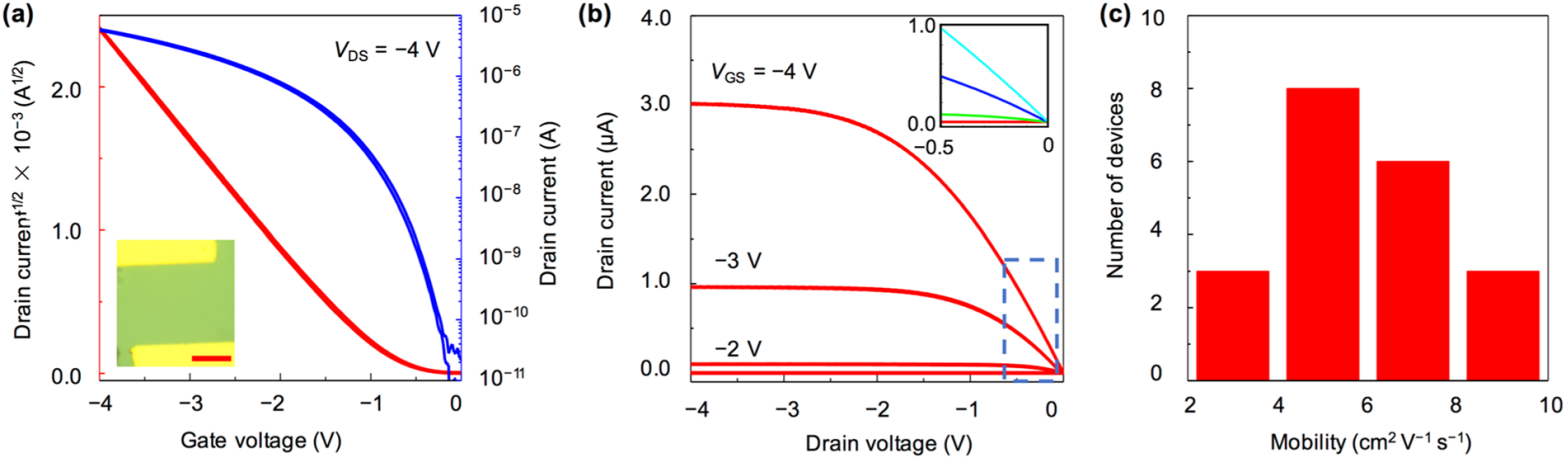
(**a**) Transfer characteristics at a drain voltage of −4 V. The inset shows the optical microscope image of the measured device. Scale bar, 20 μm. (**b**) Output characteristics at different gate voltages. Inset shows a linear increase in the drain currents in a small range of the drain voltage, indicating a nearly ideal ohmic contact in our devices. (**c**) Mobility distribution from 20 devices.

The bilayer crystalline films and AlO_x_ are used as conducting channels and gate dielectrics, respectively, to fabricate the planar transistors. Figure 3a and b show the typical transfer and output characteristics of a bilayer C_8_-BTBT-based FET, respectively. These properties indicate that a low operating voltage (−4 V) is sufficient to operate the device properly with an evident field effect. The drain current in the output curves reaches the saturation region even at a drain voltage of −4 V. A nearly linear increase in the drain current is also observed in the small range of the drain voltage, indicating a nearly ohmic contact with an efficient charge injection from the metal contact to the conducting channel (inset of Fig. 3b). Moreover, the device exhibits a high electrical performance and yields a carrier mobility of up to 6.5 cm^2^ V^−1^ s^−1^, near-zero threshold voltage of −0.7 V, small subthreshold swing of 160 mV dec^−1^, and large on/off ratio of >10^5^. The carrier mobility value was calculated in the saturation region using the equation:

1 $${I}_{D}=\,(\frac{W{C}_{i}}{2L}){\mu }_{{\rm{FET}}}{({V}_{G}-{V}_{T})}^{2}$$

where *W* and *L* are the channel width and length, respectively, *C*_i_ is the gate dielectric capacitance, *V*_T_ is the threshold voltage. Besides, 2D crystalline films with only several layers can greatly enhance the charge injection process by significantly decreasing the access resistance related to the charge injection from the metal/semiconductor interface to the active channel^27, 28^. And the width-normalized contact resistance in our device is estimated to be ~360 Ω cm by using the Y-function method; the value is among the lowest ones for the contact resistance of organic transistors (Supplementary Figs S8 and S9). For comparison, we also fabricated BGTC FETs based on a five-molecular-layer C_8_-BTBT crystal. The threshold voltage and mobility are −2.7 V and 0.98 cm^2^ V^−1^ s^−1^, respectively (Supplementary Fig. S10). Besides, the width-normalized contact resistance is 7600 Ω cm, which is much larger than that in FETs based on bilayer crystal (Supplementary Fig. S11). We observed a negligible hysteresis from the transfer curve as the applied gate voltage sweeps backwards. We also prepared 20 devices with AlO_x_ dielectrics, obtaining an average mobility of 4.7 ± 1.9 cm^2^ V^−1^ s^−1^ (Fig. 3c). Furthermore, the highest mobility obtained is 9.8 cm^2^ V^−1^ s^−1^ (Supplementary Fig. S12). To the best of our knowledge, our device exhibits a record-high value of the carrier mobility for low-voltage OFETs (Supplementary Table S2). Typical OFETs based on C_8_-BTBT reported in literature are summarized in Supplementary Table S3.

**Figure 4:**
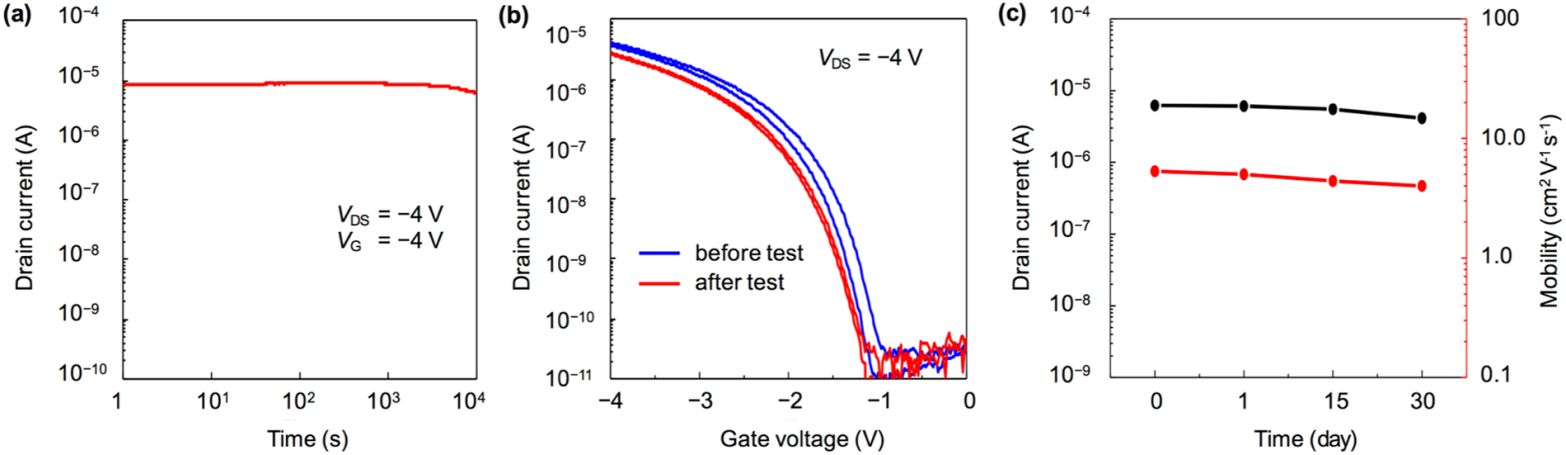
(**a**) Bias-stress characteristics of a C_8_-BTBT-based OFET. Bias-stress measurements were conducted in the ambient condition. The drain and gate voltages were both −4 V. (**b**) Transfer curves before and after the bias-stress test. (**c**) Stability characteristics of the C_8_-BTBT-based OFET.

Apart from low-voltage operation and high performance, suitable bias-stress stability is also significant for practical applications. Figure 4a shows the bias-stress characteristics of a bilayer C_8_-BTBT-based FET. Even when tested in the ambient condition, the drain current of the device shows nearly negligible change under a prolonged operation of 10^4^ s. Apart from the high-quality bilayer C_8_-BTBT crystalline films, low-voltage operation generates limited heat during the electrical measurements, hence also contributes to the stability of our transistor device. Figure 4b shows that the shapes of the transfer curves before and after the bias-stress test exhibit a small shift to a more negative gate voltage. There is a negligible change in the threshold voltage during the test (~0.1 V) (Supplementary Fig. S13). The estimated carrier mobility slightly decreases from 6.3 cm^2^ V^−1^ s^−1^ to 5.7 cm^2^ V^−1^ s^−1^. We also evaluated the stability by testing a device maintained in ambient condition for up to 30 days. Figure 4c shows that both the drain current and carrier mobility only decrease slightly.

**Figure 5:**
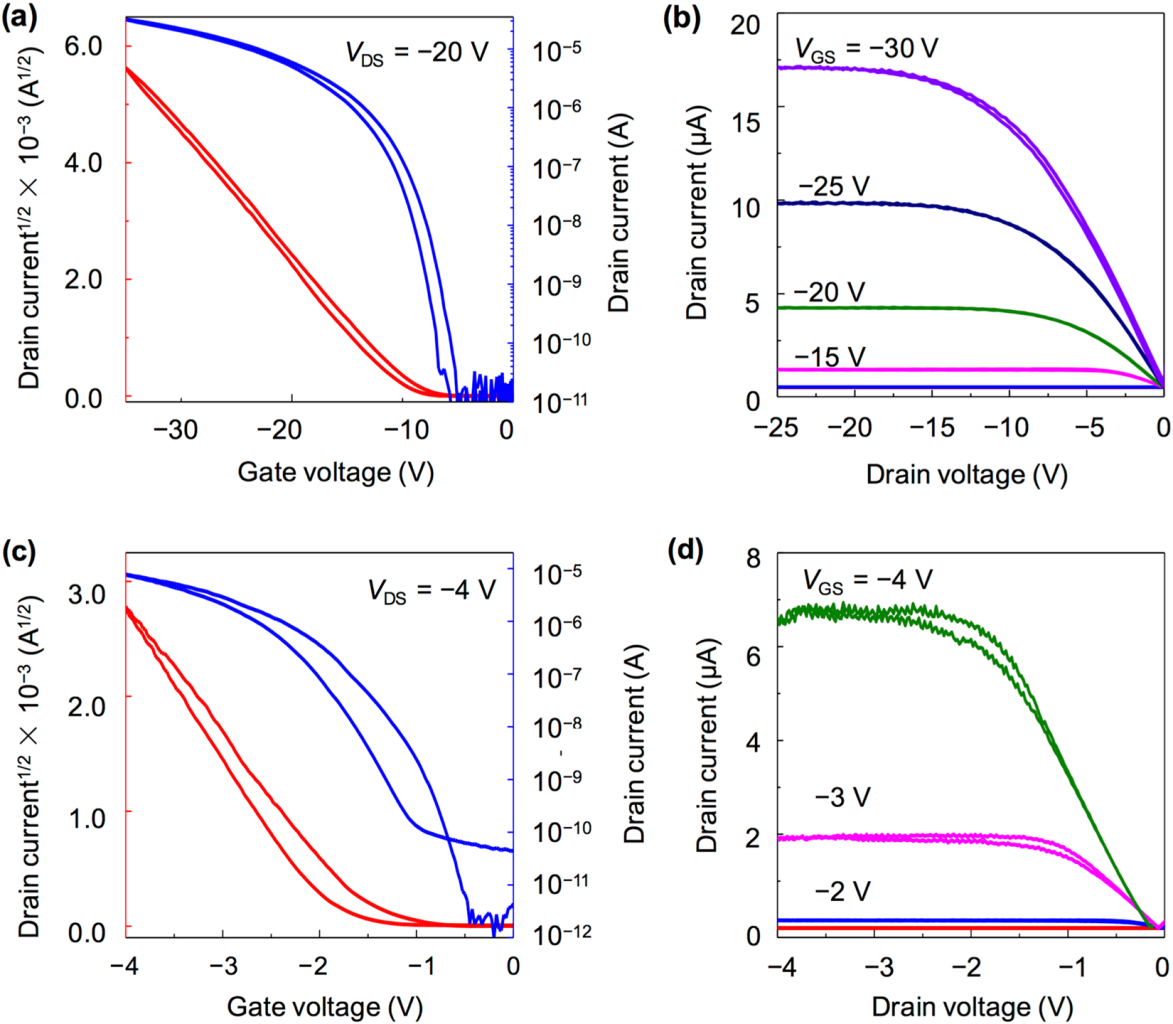
Electrical characteristics of the C_8_-BTBT-based OFETs on SiO_2_/Si and HfO_2_/Si. (**a**) Transfer characteristics at a drain voltage of −20 V. (**b**) Output characteristics at different gate voltages of a device using SiO_2_. (**c**) Transfer characteristics at a drain voltage of −4 V. (**d**) Output characteristics at different gate voltages of a device using HfO_2_.

Consequently, the presented results prove the promising features of thermally evaporated AlO_x_ as a gate dielectric for low-voltage OFETs with bilayer molecular crystals as conducting channels. For comparison, we prepared the FET samples that utilized SiO_2_ and HfO_2_ (Fig. 5). A large operating voltage of −20 V is necessary to operate the SiO_2_-based device, and the estimated carrier mobility is 4.8 ± 2.1 cm^2^ V^−1^ s^−1^. The operating voltage can be properly lowered to −4 V, whereas the carrier mobility can be as low as 0.4 ± 0.3 cm^2^ V^−1^ s^−1^ when applying HfO_2_ as the gate insulator. The decreased mobility in the device with HfO_2_ is mainly due to the strong interaction at the interface between the conducting channel and high-*κ* dielectric^29, 30^. This interaction results in the increased localization of the charge carriers^6, 31, 32^, which is consistent with the result that OFETs based SiO_2_ exhibit a higher carrier mobility than that based on AlO_x_. Further studies on the charge carrier properties in our ultrathin molecular crystals is of great interest. Thus, to develop a technique that allows for the fabrication of 2D-crystalline-film-based transistors that employ vacuum as the dielectric layer is necessary^29^.

**Figure 6:**
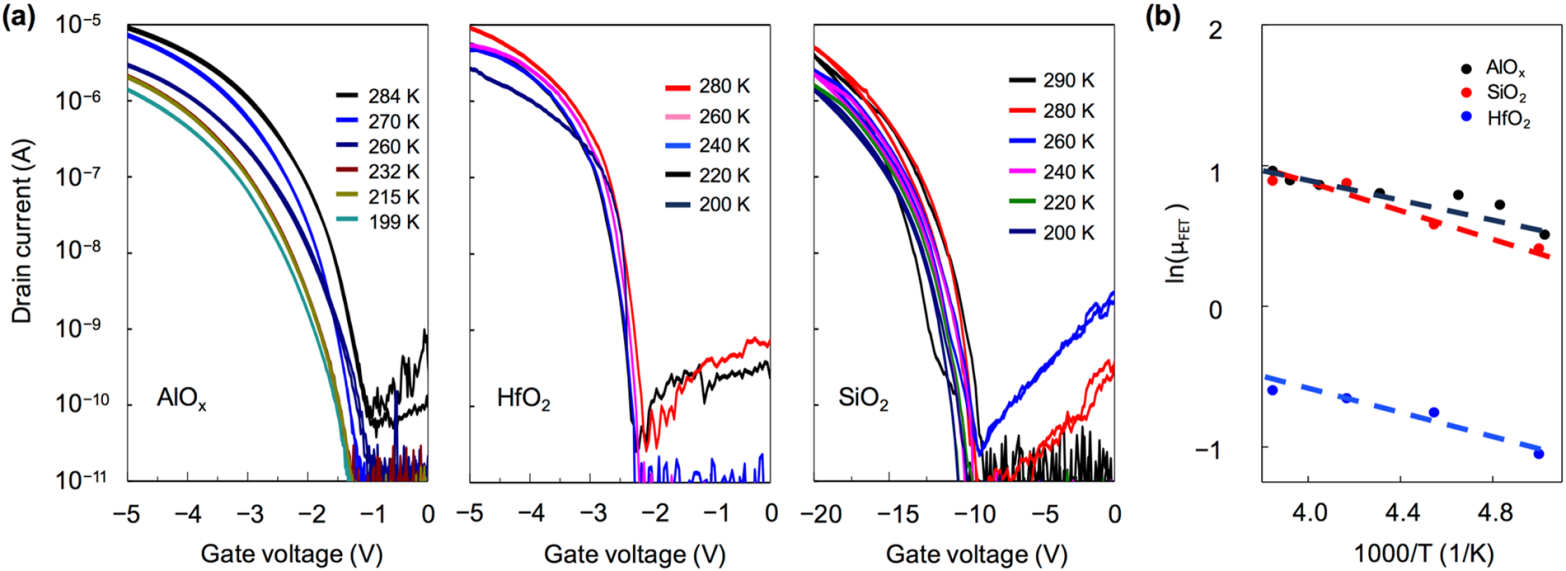
(**a**) Transfer curves of the C_8_-BTBT-based OFETs using AlO_x_, HfO_2_ and SiO_2_ under different temperatures. (**b**) Temperature dependence of the field-effect mobility.

Note that AlO_x_ also has a high dielectric constant, whereas the obtained carrier mobility is similar to that of the device with SiO_2_, and much higher than those of the HfO_2_-based devices. The maximum density of interfacial traps (*N*_trap_) is estimated from the values of the subthreshold swing (*SS*) to examine the performance, especially the carrier mobility, exhibited in the device that uses AlO_x_:

2 $${N}_{Trap}^{max}=(\frac{qSSlog(e)}{kT}-1)\frac{{C}_{i}}{q}$$

where *q* is the electronic charge, *SS* is the subthreshold swing, *e* is Euler’s number, *k* is the Boltzmann’s constant, *T* is the absolute temperature, and *C*_i_ is the gate dielectric capacitance. The trap density of AlO_x_ is ~3.9 × 10^12^ cm^−2^, which is in the same range as those of SiO_2_ and HfO_2_. The intrinsic charge transport behavior is determined by performing the temperature-dependent measurement on the electrical performance of the FETs with different oxide dielectrics (Fig. 6a). The carrier mobilities calculated from the transfer curves can all be fitted to linear lines in the plots of ln(*μ*_FET_) versus 1/*T* (Fig. 6b), which indicates that the hopping transport dominates in all devices. Furthermore, the activation energy (*E*_a_) can be estimated through the Arrhenius equation:

3 $${\mu }_{{\rm{FET}}}={\mu }_{0}exp(-{E}_{a}/kT)$$

where *μ*_0_ is the trap-free mobility. Temperature-dependent measurements for the devices with different dielectric layers are summarized in Table 1. The device that utilizes AlO_x_ exhibits the lowest *E*_a_ value of 30.8 meV and a high *μ*_0_ of 12 cm^2^ V^−1^ s^−1^. And *E*_a_ is considered to be related to the width of the distribution of trap states^33–35^. Therefore, the high carrier mobility obtained in the AlO_x_-based device is attributed to a low energetic disorder, a narrow width for the density of trap states in the dielectric interface, and a close packing among the C_8_-BTBT molecules^36–38^. The results reveal that AlO_x_ can provide a beneficial interface for the transport of charge carriers. Besides, despite a good structural quality of our bilayer C_8_-BTBT crystals and high electrical performance obtained from the AlO_x_-based transistors, the charge transport exhibits as a hopping-like rather than a band-like behavior. Similar results were also reported in literature, which imply that the property of the dielectric layer can affect the charge transport in the conducting channel^29, 38–41^. Besides, our recent results also reveal that the charge transport behavior can be greatly influenced by the contact resistance^42–44^.

**Table 1:** Electrical performances, including *μ*_FET_ (field-effect mobility), on/off ratio, *SS* (subthreshold swing), *V*_T_ (threshold voltage), maximum trap density, *E*_a_ (activation energy), and *μ*_0_ (the trap-free mobility), of the OFETs with different dielectrics.

Dielectric	*μ*_FET_ (cm^2^V^−1^s^−1^)	On/Off	*SS* (mV dec^−1^)	*V*_T_ (V)	Maximum Trap Density (×10^12^ cm^−2^)	*E*_a_ (meV)	*μ*_0_ (cm^2^ V^−1^ s^−1^)
AlO_x_	4.7 ± 1.9	10^5^	160	−0.7	3.9	30.8	12
HfO_2_	0.4 ± 0.3	10^5^	155	−1.5	4.1	36.5	4
SiO_2_	4.8 ± 2.1	10^5^	460	−9.0	2.3	39.6	16

In conclusion, we fabricated low-voltage and high-performance OFETs that employ solution-processed bilayer molecular crystals and high-*κ* material of AlO_x_ as the conducting channels and the gate dielectrics, respectively. The devices can operate under a low applied voltage of −4 V and exhibit excellent electrical performance with the highest carrier mobility of up to 9.8 cm^2^ V^−1^ s^−1^. Moreover, further studies indicated that the AlO_x_ application in FET devices is favorable to the interfaces among the 2D molecular crystals, in which the charge carrier transport has small activation energy. The results demonstrated the advantages of the proposed strategy to attain low-voltage and high-performance OFETs.

## Methods

Fabrication of the AlO_x_ layer: The Si substrate was sequentially cleaned by sonication in acetone and isopropanol for 10 min each. The oxide dielectric of AlO_x_ with a thickness of ~18 nm was thermally evaporated under a deposition speed of 0.1 Å s^−1^ with a base pressure of 10^−5^ Torr. The AlO_x_ was then treated by UV-ozone for 15 min.

Deposition of the 2D C_8_-BTBT Crystals: The p-type organic semiconductor C_8_-BTBT was supplied by Nippon Kayaku Co. and was adopted without further purification. C_8_-BTBT (1.0 wt%) was dissolved in a mixture of anisole and *p*-anisaldehyde (0.5 wt%) which were the good solvent and the antisolvent, respectively. The UV-ozone-treated AlO_x_ was sequentially cleaned in acetone and isopropanol. Before the droplet was casted onto the AlO_x_ substrate, the solution was shaken for ~30 s to deposit from a homogeneous solution. A mechanical pump was then employed to vent the air through a pipe positioned ~1 mm from the droplet (Supplementary Fig. S3).

Characterizations of the C_8_-BTBT Crystals: An Olympus BX51 was used to obtain the optical microscopy images. Two AFM types were performed in this work. The characterizations were performed on a Veeco Multimode 8 under the ambient conditions for the regular AFM. The experiments were then performed on an Asylum Cypher under ambient conditions utilizing Asylum ARROW UHF AFM tips for the high-resolution AFM.

Fabrication and Electrical Measurements of FETs: Few-layered C_8_-BTBT was deposited onto the AlO_x_ substrates for the OFET fabrication, as shown in Fig. 1. Patterned Au films with a thickness of 100 nm and Au pads with dimensions of 30 μm × 100 μm were thermally evaporated under a deposition speed of 0.2 Å s^−1^. The two Au pads were subsequently transferred to the top of the C_8_-BTBT crystal to form the source and drain electrodes (Supplementary Fig. S14). Electrical measurements were performed utilizing an Agilent B1500 semiconductor parameter analyzer in a closed-cycle cryogenic probe station with a base pressure of 10^−5^ Torr.

## Electronic supplementary material

Low-voltage, High-performance Organic Field-Effect Transistors Based on 2D Crystalline Molecular Semiconductors
