# Supplementary material for: Low-voltage, High-performance Organic Field-Effect Transistors Based on 2D Crystalline Molecular Semiconductors
Source: Sci Rep. 2017 Aug 10;7:7830. doi: 10.1038/s41598-017-08280-8 (PMC5552882; doi:10.1038/s41598-017-08280-8)
Supplement: Supplementary file 1 — Low-voltage, High-performance Organic Field-Effect Transistors Based on 2D Crystalline Molecular Semiconductors [file 41598_2017_8280_MOESM1_ESM.doc]

Supplementary Information

Low-voltage, High-performance Organic Field-Effect Transistors Based on 2D Crystalline Molecular Semiconductors

Qijing Wang^1^, Sai Jiang^1^, Jun Qian^1^, Lei Song^1^, Lei Zhang^1^, Yujia Zhang^1^, Yuhan Zhang^1^, Yu Wang^1^, Xinran Wang^1^, Yi Shi^1^, Youdou Zheng^1^ & Yun Li^1^

^1^National Laboratory of Solid-State Microstructures, School of Electronic Science and Engineering, Collaborative Innovation Center of Advanced Microstructures, Nanjing University, Nanjing 210093, China.

**Correspondence and requests for materials should be addressed to Y. L. (yli@nju.edu.cn) or Y. Shi (yshi@nju.edu.cn)**

**Figure S1.** AFM morphology images of the AlO_x_ dielectric before (left) and after (right) the UV-ozone treatment. And the RMS roughnesses are 2.23 and 2.75 Å, respectively. Scale bar, 500 nm.

**Discussion about the AlO_x_ capacitance**

Figure 1d shows the asymmetric behaviour of I-V characteristics. This might be due to the interfacial effect of Au/AlO_x_/Si capacitance structure such as the dependence of trapping-detrapping rate on the applied voltage and the resulting change of Schottky barrier.

**
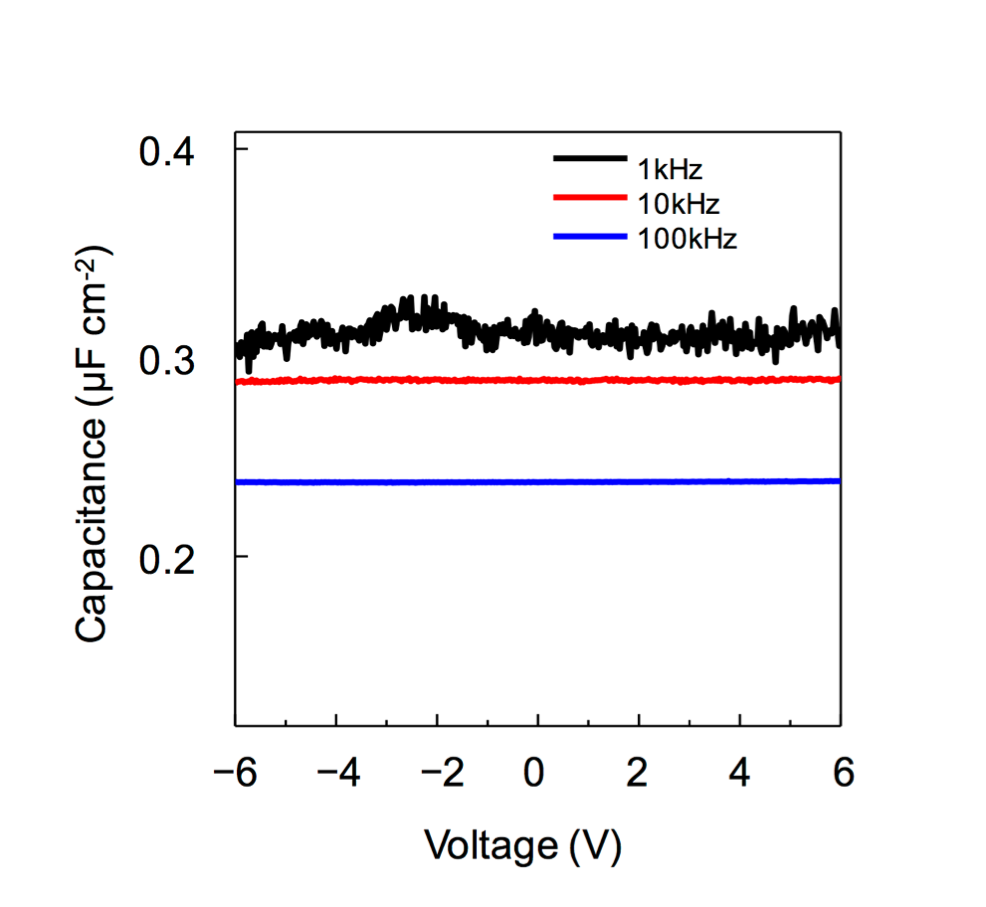
**

**Figure S2.** Capacitance per unit area (*C*_i_) verse voltage at different frequencies (1 kHz, 10 kHz, 100 kHz). The *C*_i_ is rather stable at high frequency while exhibits slight fluctuation at low frequency. Besides, an obvious decrease can be observed along with the increase in frequencies.

**Floating-coffee-ring-driven assembly**

During the deposition process, the key characteristic is the good solvent on the antisolvent layer near the droplet edge, where molecules are assembled into large-area 2D crystalline films at a high assembling rate. In the C_8_-BTBT solution with a small concentration of the antisolvent *p*-anisaldehyde, the air adjacent to the solution surface is not saturated with solvent vapour when the airflow-dragged droplet moves quickly on the substrate. A high evaporation rate near the edge and a limited amount of solution on the coating area is achieved. The differences in the boiling points and mass density of the good solvent of anisole (154 °C, 0.99 g mL^−1^) and antisolvent (258 °C, 1.22 g mL^−1^) induce a floating-coffee-ring effect of anisole on the antisolvent layer near the solution edge.

**
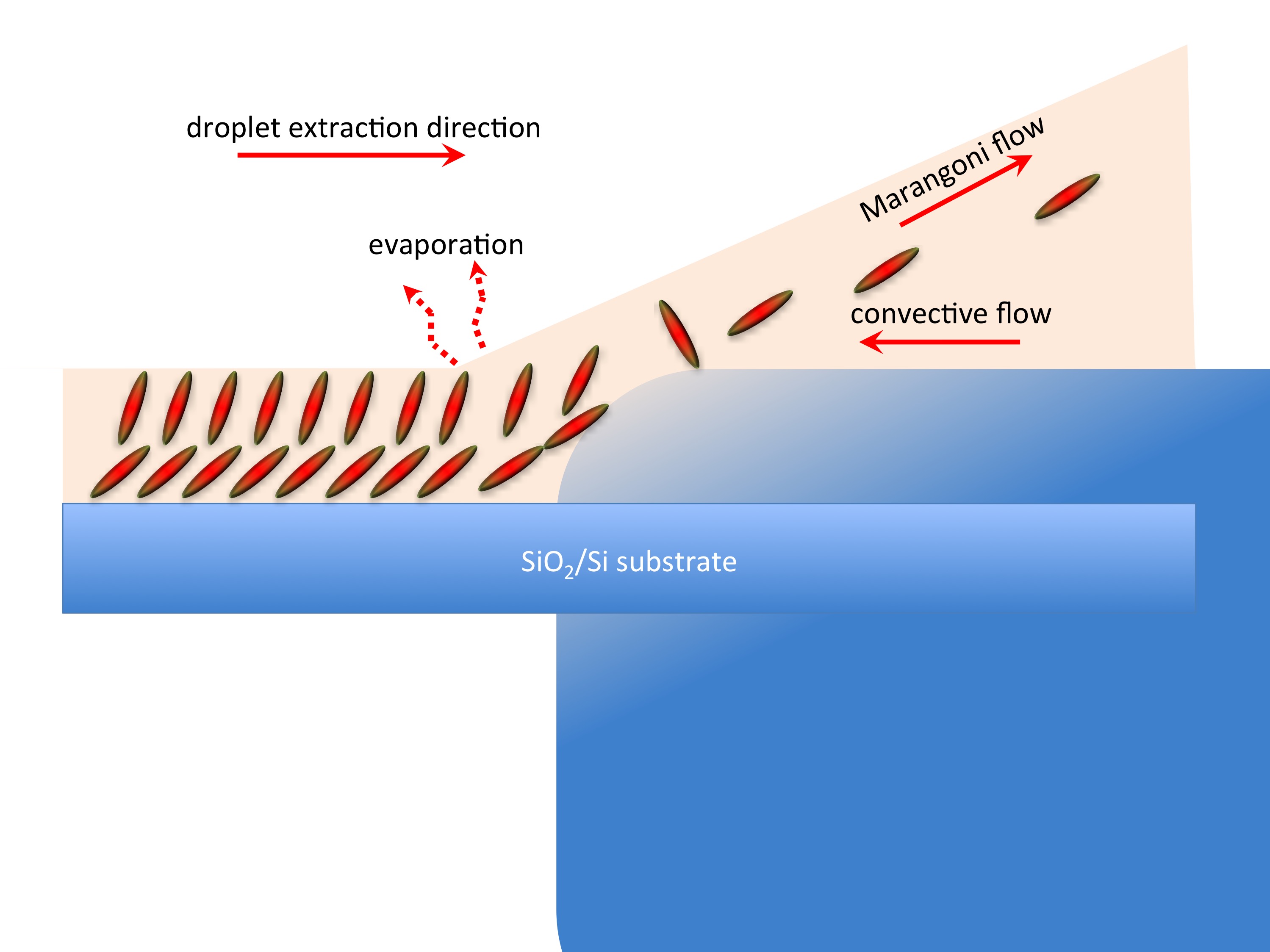
**

**Figure S3.** Schematic oft he solvent anisole fluxes in the vicinity of a growing bilayer crystalline C_8_-BTBT.

We also obtained large bilayer films with a size of several millimetres with some small multilayer domains and cracks.


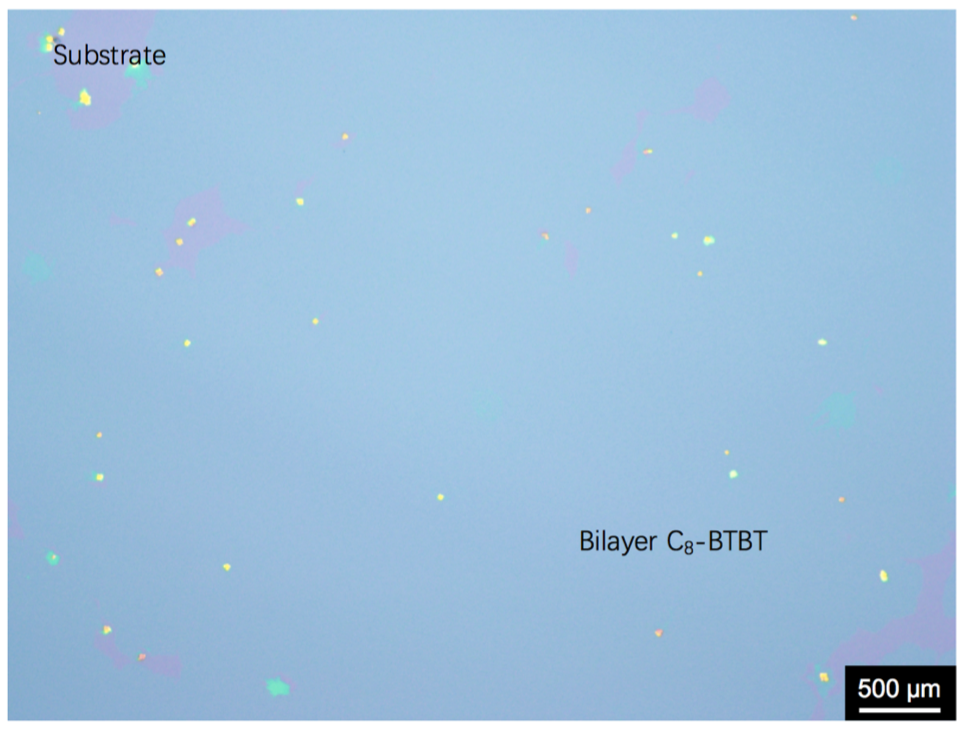


**Figure S4**. Optical microscopy image of bilayer C_8_-BTBT film

**Figure S5.** Schematic illustration of C_8_-BTBT molecular packing according to the AFM results.

**Figure S6.** Single-crystalline characterization of 2L C_8_-BTBT molecules. High-resolution AFM image (Scale bar, 1 nm) were taken from randomly chosen 10 areas in the optical microscopy image (Scale bar, 20 μm). Histogram of lattice constants for 2L C_8_-BTBT molecules is shown in the right figure.

**Figure S7.** Single-crystalline characterization of 3L C_8_-BTBT molecules. High-resolution AFM images (Scale bar, 1 nm) were taken from randomly chosen 6 areas in the optical image (Scale bar, 20 μm). Histogram of lattice constants for 3L C_8_-BTBT molecules is shown in the right figure.

**Figure S8.** Estimating the contact resistance of a transistor device with bilayer crystalline films.

We applied the Y function method (YFM) for estimating the contact resistance of a transistor device with bilayer single-crystalline C_8_-BTBT. The method was established for silicon metal-oxide-semiconductor FETs (MOSFETs)^1^ and has been applied recently in OFETs, ^2,3^ to estimate contact resistance from individual devices. In the YFM, the Y function is defined as,


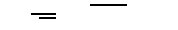
 (1)

where *G_m_* *=* *(W/L)μ_0_C_i_* is the transconductance parameter. The transconductance, *g_m_* can be written as,


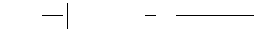
 (2)

where *θ = θ_0_+θ^*^ = θ_0_+G_m_R_sd_*. From equation (1) we obtain


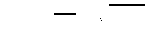
 (3)

and from equation (2)


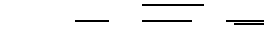
 (4)

*θ_0_* is related to surface roughness and phonon scattering, and its value is very small compared to the effective *θ* values. Thus, contact resistance can be estimated from the slopes of the plots of *Y-V_g_* and *g_m_*^−^*^1/2^-V_g_*, when the drain voltage is low to set the device in the linear regime.

To further study the application of the Y-function method, we plotted the *θ* verse the G_m_ (Figure S9). And the slope responding to the width-normalized contact resistance is 890 Ω cm, which is in the same magnitude of our data given in the manuscript. Besides, the intercept *θ*_0_ obtained here is 2.16×10^−2^ V^−1^, which is very small and negligible as compared to the effective *θ* values.

For comparison, we also fabricated BGTC FETs based on a five-molecular-layer C_8_-BTBT crystal. And the threshold voltage and mobility were −2.7 V and 0.98 cm^2^V^−1^s^−1^, respectively (Figure S10). Besides, the width-normalized contact resistance calculated by the Y-function is 7600 Ω cm, which is much larger than that in FETs based on bilayer crystal. We also obtained similar results on the devices using SiO_2_ dielectrics.

The contact resistance in a FET device contains the resistance at the metal/organic interface (interface resistance, *R*_int_) and the resistance at the access region (access resistance, *R*_acs_), where charge carriers transport from the metal/semiconductor interface to the conducting channel. Figure S11 clearly shows the difference between the width-normalized contact resistance in OFETs based on bilayer C_8_-BTBT and bulk crystals. The access resistance also has an obvious influence on the charge injection which increases the contact resistance and decrease the carrier mobility in the devices. Thus, we believe that charge injection improved by decreasing the width-normalized contact resistance is intrinsic to low-dimensional crystals. It can also be observed in 2D atomic materials.

**Figure S9.** Plot of the mobility attenuation factor with respect to the transconductance parameter.

**Figure S10.** Transfer characteristics of a transistor based on a five-molecular-layer C_8_-BTBT crystal at a drain voltage of −4V.


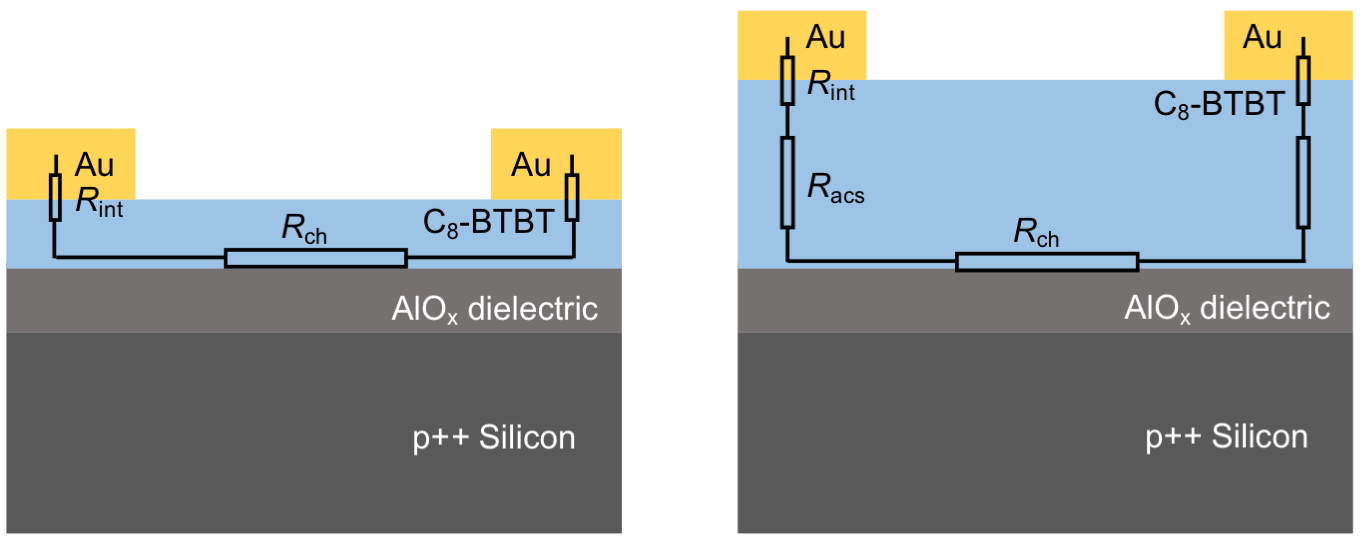


**Figure S11.** Cross-section schematic of the OFETs based on bilayer and bulk C_8_-BTBT crystals.

**Figure S12.** Transistor characteristics for the 2D crystalline C_8_-BTBT with the highest FET device performance. (a) Transfer characteristics at drain voltage of −4 V. The channel is a bilayer single-crystalline C_8_-BTBT. And a high carrier mobility of 9.8 cm^2^ V^−1^ s^−1^ was obtained. (b) Output characteristics at various gate voltage.

**Figure S13.** Square root of drain current as a function of gate voltage before and after bias-stress test, with a drain voltage of −4V.

**Au transfer technique**

During the FET devices fabrication, source and drain electrodes are transferred to the bilayer C_8_-BTBT crystalline films.

1. Patterned Au films with a thickness of 100 nm and Au pads with dimensions of 30 μm × 100 μm were thermally evaporated on the SiO_2_/Si substrates under a deposition speed of 0.2 Å s^–1^.
2. A tiny tungsten tip (tip 1) with a diameter of 1 μm is used to lift up the Au pad’s edge gently.
3. Then, another BeCu tip (tip 2) with a diameter of 15 μm, dipped with some glue, is used to lift up the Au pad off the substrate completely.
4. At last, the Au pad is transferred to the surface of bilayer C_8_-BTBT crystalline films slowly and gently.

**
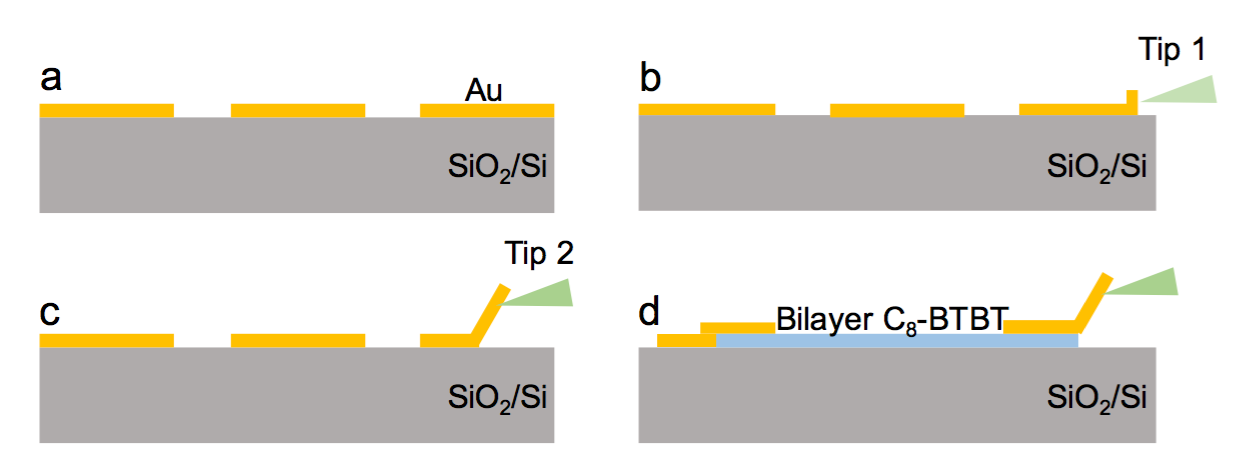
**

**Figure S14.** Cross-section schematic of the detailed procedures of Au transfer.

**Table S1.** Morphologic properties and lattice constants for 2L and 3L C_8_-BTBT molecules.

| Layer | Height  [nm] | RMS roughness  [Å] | Lattice constant | | | |
| --- | --- | --- | --- | --- | --- | --- |
|  |  |  | *a* [Å] | *b* [Å] | | *θ* [°] |
| 1+2L^a)^ | 5.21 ± 0.23 | 1.22 | 6.21 ± 0.16 | | 8.12 ± 0.12 | 88.1 ± 1.4 |
| 3L | 2.99 ± 0.19 | 1.44 | 6.16 ± 0.14 | | 8.07 ± 0.14 | 87.8 ± 1.5 |

^a)^ 1+2L means the first and second layers combined together. The height indicated the whole thickness of these two layers.

**Table S2.** Typical low-voltage OFETs in literature.

| Method | Semiconductor | Device Structure | Dielectric | Carrier Mobility  [cm^2^ V^–1^ s^–1^] | Operating Voltage  [V] | Ref. |
| --- | --- | --- | --- | --- | --- | --- |
| Solution-processed | PC12TV12T | TGBC | PMMA/P(VDF-TrFE) | 0.1 | 5 | [4] |
|  | P(NDI2OD-T2) |  |  | 0.4 | 10 |  |
| Solution-processed | pentacene | BGTC | SAM/AlO_x_ | 0.5 | 2 | [5] |
| Solution-processed | pBTTT-C16 | TGBC | P(VDF-TrFE-CFE) | 0.11 | 3 | [6] |
| Solution-processed | PBTTT-C14 | BGTC | ODPA/ZrO_x_ | 0.2 | 3 | [7] |
| Vacuum-processed | C_60_ | BGTC | pV3D3 | 1.83 | 3 | [8] |
| Solution-processed | C_2_-4T-C_12_-PA | BGTC | AlO_x_ | 10^–5^ | 4 | [9] |
|  | Glycol-C_60_-C_6_-PA |  |  | 10^–4^ |  |  |
| Solution-processed | P3HT | TGBC | phospholipid | 6.4×10^–3^ | 0.5 | [10] |
| Vacuum-processed | pentacene | BGTC | PMMA/SiO_2_ | 0.31 | 5 | [11] |
| Solution-processed | DH4T | BGBC | OTS/SiO_2_ | 0.06 | 10 | [12] |
| Vacuum-processed | DNTT | BGTC | AlO_x_ | 0.6 | 3 | [13] |
| Vacuum-processed | pentacene | BGTC | PA-SAM/HfO_2_ | 0.22 | 1.5 | [14] |
| Solution-processed | FS111 | BGTC | HfO_2_ | 1.0 | 2 | [15] |
| Solution-processed | TIPS-Pentacene/PaMS | BGBC | BST-P(VDF-HFP) | 0.14 | 1.5 | [16] |
| Solution-processed | F17-DOPF | BGTC | ODPA/AlO_x_ | 0.02 | 1.5 | [17] |
| Solution-processed | PCBM | BGTC | ODPA/AlO_x_ | 0.03 | 0.5 | [18] |
| Vacuum-processed | P3HT | TGBC | P(VPA-AA) | 0.012 | 2 | [19] |
| Solution-processed | PCBM | BGTC | HfO_2_ | 0.14 | 2 | [20] |
| Solution-processed | 1-imino nitroxide pyrene | BGTC | SiO_2_ | 0.1 | 6 | [21] |
| Solution-processed | PTAA | BGTC | TiO_2_ | 0.25 | 1 | [22] |
| Vacuum-processed | C_60_ | BGTC | AlO_x_ | 2.17 | 3 | [23] |
| Vacuum-processed | pentacene | BGTC | PVP/YO_x_ | 1.74 | 5 | [24] |
| Vacuum-processed | pentacene | BGTC | (PhO-19-PA) SAM/AlO_x_ | 1.1 | 2 | [25] |
| Vacuum-processed | pentacene | BGTC | PMMA/Ta_2_O_5_ | 0.4 | 1 | [26] |
| Solution-processed | P3HT | BGTC | ZrO_2_ | 0.08 | 3.5 | [27] |
| Vacuum-processed | pentacene | BGTC | P*a*MS/ZrO_2_ | 0.9~1.2 | 3 | [28] |
| Vacuum-processed | pentacene | BGTC | SAM/AlO_x_ | 1 | 3 | [29] |
| Vacuum-processed | DNTT | BGTC | SAM/AlO_x_ | 3 | 5 | [30] |
| Solution-processed | C_8_-BTBT | BGTC | AlO_x_ | 9.8 | 4 | **our work** |

**Table S3.** Typical OFETs based on C_8_-BTBT in literature.

| Method | Device structure | Dielectric | Average  Mobility  [cm^2^V^−1^s^−1^] | Maximum mobility  [cm^2^V^−1^s^−1^] | Operating Voltage  [V] | Ref. |
| --- | --- | --- | --- | --- | --- | --- |
| Solution-processed | BGTC | PVP | 25 | 43 | 40 | [31] |
| Solution-processed | TGTC | Parylene C | 16.4 | 31.3 | 60 | [32] |
| Solution-processed | BGTC | SiO_2_/PMMA | 3 | 9.1 | 40 | [33] |
| Solution-processed | BGTC | SiO_2_/PS |  | 3.56 | 40 | [34] |
| Solution-processed | TGBC | CYTOP | 9.4 | 13 | 80 | [35] |
| Vacuum-processed | BGTC | SiO_2_/ODTS |  | 2.9 | 60 | [36] |
| Vacuum-processed | TGBC  TGTC | CYTOP | 5.5~5.7 |  | 40 | [37] |
| Solution-processed | TGBC | CYTOP/Polymer | 7.9 |  | 40 | [38] |
| Solution-processed | BGTC | SiO_2_/PMMA | 3 | 9.1 | 40 | [39] |
| Solution-processed | BGTC | SiO_2_/DTS | 4~6 |  | 100 | [40] |
| Solution-processed | BGTC | SiO_2_/OTS | 9.2 | 10.4 | 60 | [41] |
| Solution-processed | BGTC | SiO_2_/FTS | 0.2~3.5 | 3.5 | 40 | [42] |
| Solution-processed | BGTC | SiO_2_/OTS | 2.6 | 6.9 | 40 | [3] |
| Solution-processed | TGBC | CYTOP | 1.59 | 2.6 | 60 | [43] |
| Vacuum-processed | BGTC | SiO_2_ |  | 2.3 | 20 | [44] |
| Solution-processed | BGTC | SiO_2_/PMMA |  | 16 | 40 | [45] |
| Vacuum-processed | BGTC | SiO_2_/BN |  | 10 | 40 | [46] |
| Solution-processed | BGTC | SiO_2_/HMDS | 0.4 | 0.7 | 60 | [47] |
| Solution-processed | BGTC | SiO_2_/PMMA | 1.1 | 3.8 | 40 | [48] |
| Solution-processed | BGTC | SiO_2_ | 4.8 | 13 | 20 | [49] |
| Solution-processed | BGTC | AlO_x_ | 4.7 | 9.8 | 4 | This work |

**References:**

(1) Ghibaudo, G. *et al.* New method for the extraction of MOSFET parameters. *Electronic Letters* **24**, 543–545 (1988).

(2) Xu, Y. *et al.* Direct Evaluation of Low-Field Mobility and Access Resistance in Pentacene Field-Effect Transistors. *Journal of Applied Physics* **107**, 114507–114507 (2010).

(3) Li, Y. *et al.* Patterning Solution-Processed Organic Single-Crystal Transistors with High Device Performance. *AIP Advances* **1,** 022149–8 (2011).

(4) Baeg, K.-J. *et al.* Low-Voltage, High Speed Inkjet-Printed Flexible Complementary Polymer Electronic Circuits. *Organic Electronics* ***14*,** 1407–1418 (2013).

(5) Huang, T.-C. *et al.*  Pseudo-CMOS: a Design Style for Low-Cost and Robust Flexible Electronics. *IEEE Trans. Electron Devices*, **58,** 141–150 (2016).

(6) Li, J. *et al.* Solution Processable Low-Voltage Organic Thin Film Transistors with High-K Relaxor Ferroelectric Polymer as Gate Insulator. *Adv. Mater.* **24,** 88–93 (2011).

(7) Park, Y. M. *et al.* Room-Temperature Fabrication of Ultrathin Oxide Gate Dielectrics for Low-Voltage Operation of Organic Field-Effect Transistors. *Adv. Mater.* **23,** 971–974 (2011).

(8) Moon, H. *et al.* Synthesis of Ultrathin Polymer Insulating Layers by Initiated Chemical Vapour Deposition for Low-Power Soft Electronics. *Nat. Mater*. **14,** 628–635 (2015).

(9) Novak, M. *et al.* Influence of Self-Assembled Monolayer Dielectrics on the Morphology and Performance of Α,Ω-Dihexylquaterthiophene in Thin Film Transistors. *Appl. Phys. Lett.* **98,** 093302–093303 (2011).

(10) Cotrone, S. *et al.* Phospholipid Film in Electrolyte-Gated Organic Field-Effect Transistors. *Organic Electronics* **13,** 638–644 (2012).

(11) Li, L.; *et al.* Controllable Growth and Field-Effect Property of Monolayer to Multilayer Microstripes of an Organic Semiconductor. *J. Am. Chem. Soc.* **132,** 8807–8809 (2010).

(12) Leydecker, T. *et al.* Solution-Processed Field-Effect Transistors Based on Dihexylquaterthiophene Films with Performances Exceeding Those of Vacuum-Sublimed Films. *ACS Appl. Mater. Interfaces* **6,** 21248–21255 (2014).

(13) Zschieschang, U. *et al.* Flexible Low-Voltage Organic Transistors and Circuits Based on a High-Mobility Organic Semiconductor with Good Air Stability. *Adv. Mater.* **22,** 982–985 (2009).

(14) Acton, O.; *et al.* Π-Σ-Phosphonic Acid Organic Monolayer/Sol-Gel Hafnium Oxide Hybrid Dielectrics for Low-Voltage Organic Transistors. *Adv. Mater.* **20,** 3697–3701 (2008).

(15) Tetzner, K. *et al.* Photonic Curing of Sol–Gel Derived HfO2 Dielectrics for Organic Field-Effect Transistors. *Ceramics International* **40,** 15753–15761 (2014).

(16) Faraji, S. *et al.* Solution-Processed Nanocomposite Dielectrics for Low Voltage Operated OFETs. *Organic Electronics* **17,** 178–183 (2015).

(17) Ball, J. M. *et al.*Complementary Circuits Based on Solution Processed Low-Voltage Organic Field-Effect Transistors. *Synthetic Metals* **159,** 2368–2370 (2009).

(18) Ball, J. M. *et al.* Solution Processed Low-Voltage Organic Transistors and Complementary Inverters. *Appl. Phys. Lett.* **95,** 103310–103313 (2009).

(19) Herlogsson, L. *et al.* Low-Voltage Polymer Field-Effect Transistors Gated via a Proton Conductor. *Adv. Mater.* **19,** 97–101 (2007).

(20) Tiwari, S. P. *et al.* Low-Voltage Solution-Processed N-Channel Organic Field-Effect Transistors with High-K HfO2 Gate Dielectrics Grown by Atomic Layer Deposition. *Appl. Phys. Lett.* **95,** 223303–223304 (2009).

(21) Wang, Y. *et al.* 1-Imino Nitroxide Pyrene for High Performance Organic Field-Effect Transistors with Low Operating Voltage. *J. Am. Chem. Soc.* **128,** 13058–13059 (2006).

(22) Majewski, L. A. *et al.* Low-Voltage, High-Performance Organic Field-Effect Transistors with an Ultra-Thin TiO2 Layer as Gate Insulator. *Adv. Funct. Mater.* **15,** 1017–1022 (2005).

(23) Zhang, X.-H. *et al.* Low-Voltage Flexible Organic Complementary Inverters with High Noise Margin and High Dc Gain. *Appl. Phys. Lett.* **94,** 043312–043313 (2009).

(24) Hwang, D. K. *et al.* Low-Voltage High-Mobility Pentacene Thin-Film Transistors with Polymer/High-K Oxide Double Gate Dielectrics. *Appl. Phys. Lett.* **88,** 243513–243514 (2006).

(25) Hutchins, D. O. *et al.* Solid-State Densification of Spun-Cast Self-Assembled Monolayers for Use in Ultra-Thin Hybrid Dielectrics. *Applied Surface Science* **261,** 908–915 (2012).

(26) Deman, A. L. *et al.* PMMA–Ta2O5 Bilayer Gate Dielectric for Low Operating Voltage Organic FETs. *Organic Electronics* **6,** 78–84 (2005).

(27) Beaulieu, M. R. *et al.* Solution Processable High Dielectric Constant Nanocomposites Based on ZrO 2Nanoparticles for Flexible Organic Transistors. *ACS Appl. Mater. Interfaces* **5,** 13096–13103 (2013).

(28) Zirkl, M. *et al.* Low-Voltage Organic Thin-Film Transistors with High-K Nanocomposite Gate Dielectrics for Flexible Electronics and Optothermal Sensors. *Adv. Mater.* **19,** 2241–2245 (2007).

(29) Klauk, H. *et al.* Ultralow-Power Organic Complementary Circuits. *Nature* **445,** 745–748 (2007).

(30) Kaltenbrunner, M. *et al.* An Ultra-Lightweight Design for Imperceptible Plastic Electronics. *Nature Publishing Group* **499,** 458–463 (2016).

(31) Yuan, Y. *et al.* Ultra-High Mobility Transparent Organic Thin Film Transistors Grown by an Off-Centre Spin-Coating Method. *Nature Communications* **5,** 1–9 (2014).

(32) Minemawari, H. *et al.* Inkjet Printing of Single-Crystal Films. *Nature Publishing Group* **475,** 364–367 (2011).

(33) Liu, C.; *et al.* Solution-Processable Organic Single Crystals with Bandlike Transport in Field-Effect Transistors. *Adv. Mater.* **23,** 523–526 (2010).

(34) Huang, Y. *et al.* Controllable Thin-Film Morphology and Structure for 2,7-Dioctyl[1]Benzothieno[3,2-B][1]Benzothiophene (C8BTBT) Based Organic Field-Effect Transistors. *Organic Electronics* **36,** 73–81 (2016).

(35) Paterson, A. F. *et al.* Small Molecule/Polymer Blend Organic Transistors with Hole Mobility Exceeding 13 Cm 2V −1s −1. *Adv. Mater.* **28,** 7791–7798 (2016).

(36) Izawa, T. *et al.* Molecular Ordering of High-Performance Soluble Molecular Semiconductors and Re-Evaluation of Their Field-Effect Transistor Characteristics. *Adv. Mater.* **20,** 3388–3392 (2008).

(37) Darmawan, P. *et al.* Optimal Structure for High-Performance and Low-Contact-Resistance Organic Field-Effect Transistors Using Contact-Doped Coplanar and Pseudo-Staggered Device Architectures. *Adv. Funct. Mater.* **22,** 4577–4583 (2012).

(38) Minari, T. *et al.*  Room-Temperature Printing of Organic Thin-Film Transistors with Π-Junction Gold Nanoparticles. *Adv. Funct. Mater.* **24,** 4886–4892 (2014).

(39) Minari, T. *et al.*  Controlled Self-Assembly of Organic Semiconductors for Solution-Based Fabrication of Organic Field-Effect Transistors. *Adv. Mater.* **24,** 299–306 (2011).

(40) Soeda, J. *et al.* Solution-Crystallized Organic Field-Effect Transistors with Charge-Acceptor Layers: High-Mobility and Low-Threshold-Voltage Operation in Air. *Adv. Mater.* **23**, 3309–3314 (2011).

(41) Kwon, S. *et al.* Organic Single-Crystal Semiconductor Films on a Millimeter Domain Scale. *Adv. Mater.* **27**, 6870–6877 (2015).

(42) Xu, C.; *et al.* A General Method for Growing Two-Dimensional Crystals of Organic Semiconductors by “Solution Epitaxy.” *Angew. Chem.* **128**, 9671–9675 (2016).

(43) Endo, T. *et al.*  Solution-Processed Dioctylbenzothienobenzothiophene-Based Top-Gate Organic Transistors with High Mobility, Low Threshold Voltage, and High Electrical Stability. *Appl. Phys. Express* **3**, 121601–121603 (2010).

(44) Kano, M. *et al.* Improvement of Subthreshold Current Transport by Contact Interface Modification in P-Type Organic Field-Effect Transistors. *Appl. Phys. Lett.* **94**, (143304–3 (2009).

(45) Cho, J.-M. *et al.* Band-Like Transport Down to 20 K in Organic Single-Crystal Transistors Based on Dioctylbenzothienobenzothiophene. *Appl. Phys. Lett.* **106**, 193303–193304 (2015).

(46) He, D. *et al.* Two-Dimensional Quasi-Freestanding Molecular Crystals for High-Performance Organic Field-Effect Transistors. *Nature Communications* **5**, 1–7 (2017).

(47) Wang, Y. *et al.* Solution-Processed Organic Crystals Written Directly with a Rollerball Pen for Field-Effect Transistors. *Organic Electronics* **15**, 2234–2239 (2014).

(48) Kumatani, A. *et al.* Solution-Processed, Self-Organized Organic Single Crystal Arrays with Controlled Crystal Orientation. *Sci. Rep.* **2**, 2411–2416 (2012).

(49) Wang, Q. *et al.* 2D Single-Crystalline Molecular Semiconductors with Precise Layer Definition Achieved by Floating-Coffee-Ring-Driven Assembly. *Adv. Funct. Mater.* **26**, 3191–3198 (2016).
